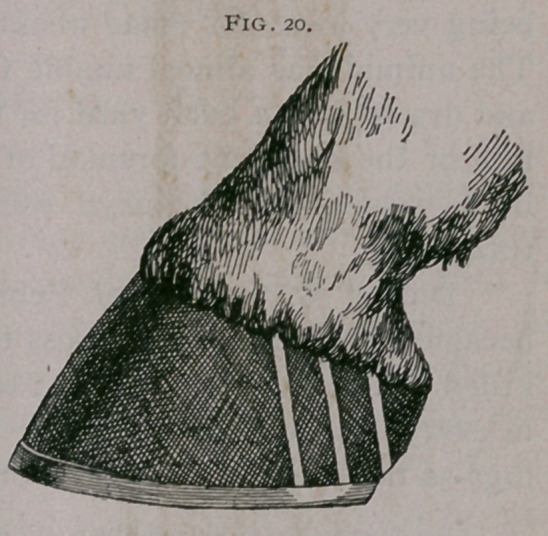# Contraction of the Horse’s Foot1Read before the Twenty-seventh Annual Meeting of the United States Veterinary Medical Association at Chicago, Sept. 17th, 1890.

**Published:** 1890-10

**Authors:** Rush Shippen Huidekoper

**Affiliations:** Veterinarian


### CONTRACTION OF THE HORSE'S FOOT.[1]

BY RUSH SHIPPEN HUIDEKOPER, M.D., VETERINARIAN.

Syn.—*Zwanghuf*, German; *Encastelure*, French; *Incastellatura*, Italian; *Encatenadura*, Spanish; *Hoof-bound*, *Contracted Heels*, English.

THE aphorism of "no foot, no horse" has been used for years in such a general way, conveying so many varied ideas to the hearer, that it has become sententious for the veterinarian to use it to the outside world; it is too accustomed to its daily reiteration from quack farriers and conceited horsemen who have personal theories, each of their own, which, having been derived from some discovery in regard to *a* horse's foot and resulted in benefit to *that* horse, is supposed and claimed to be applicable to *all* horses' feet. They know that the horse's foot is more or less intricate in its structure, but look on it as an important article much as they would upon a patent collar, or toe weight which has given a good result in one case, and therefore they suppose it should in all. Before comparative anatomists and practical veterinarians, however, no aplogy is needed for reviewing their knowledge of the foot, or adding any detail, however small, to it.

The anatomist, who might have been a Darwin or a Leidy of his race, ages ago, when the four-footed animals first came upon the earth could certainly never have conceived that the free moving ten bones of the carpus or tarsus, and the multiple digits of the animals of their day, could be diminished to a single toe, and it serve as a useful member, finding support only by its distal extremity. We, as practitioners, are too apt to overlook the importance which the remnants of the evolution in the horse's foot bears on its practical troubles and diseases, and we too often fall into the habit of regarding the feet of one horse as structures identical with those of another, which they are not.

For convenience in this paper, the word foot will be used, as meaning the "surgical foot" from the second phalanx and coronary band down, and will be qualified as the anatomical foot, if it is so meant. While the elements of the feet are the same in all the solipeds, it is important to review some of the anatomical

[2] Read before the Twenty-seventh Annual Meeting of the United States Veterinary Medical Association at Chicago, Sept. 17th, 1890.

and physiological peculiarities which allow the shape of a thoroughbred's foot to appear like a cylinder, while that of a Clydesdale has the conformation of a flat cone.

Comparing the two third phalanges, it is seen that the transverse diameter of one from a flat-footed horse is much greater in relation to its antero-posterior and supero-inferior diameter, that the concavity of the plantar surface is much less, and that the basilar and retrossal processes stand out at an angle from the median line of the bone instead of lying almost parallel to it, as they do in the bone from a mule-footed horse. In the flat foot the articulation of the third phalanx with the second phalanx above is more shallow, and consequently admits of a greater amount of lateral motion between the two bones. The difference between the navicular bones of the two feet is only one of accommodation, except that in the flat foot the navicular is closer to the plantar surface and has greater freedom of movement. We recognize that the ligaments of flat feet have greater surfaces of attachment, and in the lymphatic animal are less dense and more extensible, thereby allowing a greater freedom of movement, but predisposing to a greater amount of bone irritation if they are wrenched at their origin on the bone. The plantar cushion of the flat foot is larger in proportion to the size of the foot than in the high-heeled foot, as in the former there is a greater divergence of the wings from the median line of the pedal bone. The walls accommodate themselves to the difference of relationship of the internal parts. The flat foot, in profile, is seen truncated at a more acute angle. The greater and wider frog, sunk at the glomes of the heels nearer the ground, renders in some cases the entire posterior part of the foot a soft cushion, holding up both quarters and bar, whereas, in feet of the opposite conformation, these latter must be considered as the important factors in the movement of the heels, while the frog plays a secondary part, acting as a passive support. In any horse the fore and hind feet differ slightly, the former always approaching more to the type of the flat foot, while the hind ones resemble more those of the high-heeled foot.

For the purpose of this paper, the physiology of the foot may be reduced to the recognition of a solid bony centre (second and third phalanx and navicular bone), with elastic lateral wings (lateral fibro-cartilages), surrounded by a vascular network admitting of moderate movement, (*rete processigerum*, *rete plantarum*, and synovials of the flexor tendons), which rest on an elastic cushion, and are enveloped in a dense keratogenous box (wall, sole, and frog) which plays only a passive part in the functional activity of the whole. The internal parts are rich in nutrient bloodvessels, and are luxuriant in nerves (tactile, general sensation and trophic).

The keratogenous covering should be dense and firm, to resist friction, and moist and pliable, to accommodate itself to alterations of pressure.

The elasticity and distensibility of the horse's foot was recognized by Solleysel, and was made the object of special study by Bracy-Clark, Coleman, Gloag and Bouley, all of whom left information of value.

Professor J. Lechner, of Vienna,[1] was one of the leaders in the study of the relative movements of the various parts of the foot, and he formulated a theory that, when pressure is placed on the foot from above, there is a dilatation of the coronary portion with a retraction of the plantar edge of the heels, producing a rotation of the posterior extremity of the walls. Professor Lechner employed in his experiments several instruments, one of the most ingenious of which was an electric current and bell. (Fig. 1). A rod (*a*) fastened to the toe of the horse's foot, as this is the most fixed point, follows the line of the wall to the heel and holds a pin (*b*) which can be screwed in and out; the latter faces a piece of zinc set in the quarter (*c*); from the pin and zinc (*b* and *c*) wires (*dd*), extend to a bell. When weight is placed on the foot and the heels expand, communication is established between zinc and pin, and, the current connected, notice is given of it by the bell. The amount of expansion could be determined by a graduation on the pin. Professor Lechner's paper elicited considerable controversy, and his work was followed by numerous experiments and studies by others.

FIG. 1.

Lungwitz and Schaaf, by means of an ectasimeter of their own, arrived at the following conclusions:

[1] Ueber Hufrotation. Vortrag gehalten in der Section XI Veterinärkunde) der 54 versammlung deutcher Naturforscher und Ærtze zu Salzburg, 1881

1st. The raised foot is smaller at the plantar surface than it is when upon the ground.

2d. Dilatation of the plantar border augments with the velocity of the gait.

3d. The inside heel dilates more than the outside one.

4th. The heels of the hind feet dilate less than those of the fore feet in the same horse.

5th. The coronary band dilates at the same time as the plantar border.

6th. Healthy or diseased feet dilate above, under pressure.

7th. The dilatation of the posterior part of the foot causes a shortening of the foot. (Fig. 2).

8th. The dilatation of the plantar part of the hoof varies with the form of the foot. In angular, narrow feet it increases toward the quarters and diminishes toward the heels.

9th. Normal dilatation is not complete in shod feet.

10th. Suppleness of the horn and integrity of the frog are necessary for normal and complete dilatation of the foot.

FIG. 2.

Pedal bone.

*a.* Circle.

*á.* Expanded laterally and shortened.

11th. Shoeing and dryness of the horn interfere with the movements of the foot.

12th. In contracted heels there is a certain dilatation of the quarters, but the heels not only do not take part in the dilatation, but, on the contrary, retract slightly.

FIG. 3.

*a.* Line of pressure; *b.* Schematic plantar cushion; *c.* Same, expanded; *d.* Wall unexpanded; *e.* Convex sole; *f.* Wall expanded.

Prof. Bayer, of Vienna, Martinak, of Prague, by means of a circular compass, and Dr. Schwentsky obtained about the same results.

Steglich decided that the coronary band and the plantar border dilate simultaneously, but that the former dilates the most from the pressure of the second phalanx, while the dilatation of the plantar border is due to the expansion of the plantar cushion and the flattening of the sole. (Fig. 3).

Peters, of Schwerin, from anatomo-mechanical deductions, claimed the expansion to be due to a circular trajectory of the pedal bone. (Fig. 4). This, of course, included pressure on the elastic cushions and the convex upper surface of the sole, as in the experiments of Steglich. Dominick, by experiments on the dead foot, arrived at the same conclusions.

Foringer carried out his investigations by applying a solid shoe having vertical arms on its branches, which held movable screws; his experiments were performed on the natural foot. (Fig. 5).

FIG. 4.

*a*. Without pressure; *b*. With pressure.

Adams, of Augsburg, with the same instrument, determined the dilatation of the normal foot to be three millimetres at the plantar border and one millimetre at the coronary band; he repeated and verified his work with other instruments. Some twenty other experimenters arrived at analogous conclusions.

From the evidence of these investigators we accept as proven

FIG. 5.

that the quarters and heels of the normal foot of the horse dilate under pressure, that is, when the animal is standing, and more so when it places increased weight on the foot in movement. I, however, agree with Lechner as to the rotation of the heels in some feet, and find that in contracted feet, this rotation is an important factor in increasing the disease. Lechner possibly experimented on slightly contracted high-heeled horses. We can conclude that anything which interferes with the dilatability of the hoof produces an abnormal condition, and must interfere with the vascular structures and nerves contained inside, producing atrophy of the tissues from diminished nutrition, and pain from pressure on the nerves. When any interference with the dilatation of the foot is permanent, it produces a diminution in the size of the organ. This reduction in size may be general, including the whole foot, or local, including only one or both heels ; this condition is known as *contraction of the foot, or contracted heels.*

*Contraction of the foot*, or *contracted heels*, is evidenced by a diminution in the size of the horny covering of the foot or the hoof, with or *without* lameness. Contraction may be total or partial ; in the first case, there is seen to be a general diminution o size, usually accompanied by an increased concavity of the sole, an atrophy at the frog and an approach of the walls of the quarters and heels toward a more vertical position ; the surface of the wall is frequently excessively dry, and predisposed to superficial ridges or cracks ; in partial contraction the alteration is usually confined to the heels. Contraction may be symmetrical or unilateral ; in the former case, both sides being equally affected, there is usually total diminution in the size of the foot, while in the latter the alteration in shape is generally local and more easily remedied. The older veterinarians used the terms *true* and *false* to designate a general or limited alteration in shape. True or total contraction, approaching in form the mule foot, narrowed from side to side, with vertical walls, a natural condition in the ass and mule, in the horse may be an *acquired* condition, in which case it is usually incurable, or it may be a congenital condition, in which the contents have been formed, adapted to the altered circumstances ; this can hardly be considered as morbid, but while it may not be a diseased foot in itself, it predisposes to all the other troubles which are found, the result of contraction from any other cause.

### ETIOLOGY.

The study of the etiology of contraction of the feet is, in some cases, an easy matter, but in others it is complicated by various troubles, so that it becomes difficult to determine the cause, or to distinguish in from the effect. In the majority of cases the secondary effects, or sequelæ, of contraction of the feet are troubles, which, if they had occurred in a sound leg, would have caused contraction of the foot as a complicating disease.

*Race* has for a long time been recognized as a predisposing cause, and contraction certainly occurs more frequently in horses of breeds which have thick, hard, rapidly-growing hoofs, of dense structure, than those with hoof walls of a more delicate structure. The Oriental horse has been accused, but I believe somewhat unjustly, of being prone to this affection.

*Heredity* is an important etiological factor. The horse with feet predisposed to contraction will get progeny with like feet; the horse who has, from any cause, acquired contracted feet, is apt to transmit the anatomical defect to his get, and such an animal, although he may not be lame, should be excluded from the stud.

*Dry climate* and *Summer weather* tend to draw the natural moisture from the horny structures, diminish their elasticity and favor retraction of the tissues, which ends in permanent contraction. Dryness is more serious when alternating with moisture than it is on animals who have been reared in such a dry climate, as the horse of the desert, whose constitution has accommodated itself to its surroundings.

*Stabulation* affects the hygroscopicity of the hoof to a marked degree, which is increased when it is continuous for several days at a time, alternating with exposure to excessive moisture. Under these conditions the fluids of the horny tissue do not seem to be able to find or retain their normal relations to it. An example of the effect of constant dryness is seen in the dead hoof, which alters its shape even when filled with plaster-of-Paris.

*Inaction* of the animal, a result of stabulation, diminishes the moisture of the hoof, as it slows the current in the vessels and reduces the amount of blood pressure on its inner surface.

*Too long continuance of the shoes* and *want of dressing of shod feet* produce the same result by elongating the tissues from their vascular supply. In a stallion, the subject of a legal controversy, kept for twelve months in a stall, the overgrown hoof diminished to one-half its natural diameter, and curled up like the horns of a ram.[1]

*Rasping* the walls of the foot after shoeing favors evaporation, and diminishes the hygroscopic power of the horn. *Hot shoes*,

[1] Specimen in museum at veterinary school, Alfort, France.

which evaporate the fluids from the horn, render the latter improper to reabsorb and hold moisture.

Mechanically, contraction is frequently produced by the vicious system, so common in some shops, of "opening the heels," or cutting away the bars, which are the natural support of the heels and quarters. Shoes fitted too tightly to the heels, so as to hold them and prevent their natural expansion, and nails driven too far back on the quarters, as is seen in the shoeing of some race-horses, both serve as starting points for subsequent contraction.

*Pain*, whether in the foot, or in other parts of the leg, from

FIG. 6.

Schematic Column. First, Second and Third Phalanges.

I, Normal; II, Uneven paring, producing at *a* pressure on the bones, at *b* strain of ligaments; III, A ringbone, producing pressure on quarter, same side.

contraction itself, corns, quittors, wounds, ringbones or trouble in tendons, when it causes the animal to rest its leg and ease the foot from the ground, produces inaction, want of function, diminished circulation and exposure to the evaporating currents of air, and is a frequent cause of the disease. Traveling at speed on hard roads and certain other demands, which may only cause temporary pain in the foot, may, at the same time, be the starting point of contraction.

*Lateral* deviation of the foot from its normal position, whether the cause be in the foot itself, in the pastern, or higher up, at the fetlocks, brings pressure on the sides of a quarter, forces the latter in and exposes the other quarter, and quickly produces trouble. Uneven paring of the foot and crooked shoeing, quittors and ringbones, in this way are frequent causes.

Surgical operations for quittor and pricked foot are frequently followed by contraction.

SYMPTOMS.

The characteristic appearance of a contracted foot is usually sufficient to allow of its recognition. There is an alteration in form, which may be total or partial. In the former case the foot is smaller than its fellow (if both feet are contracted, it is rare that they are of equal size); the foot is ovoid, from the diminished quarters and seemingly increased antero-posterior diameter; the heels are high, or, in flat feet, may be found with their outer walls lying on the branches of the shoe. The frog is atrophied, and exudes a foul-smelling sweat from the lacunæ, and the concavity of the sole is increased. The wall is found dry and hard, or at times has a peculiar shiny appearance, and it is frequently lined with little fibrillar cords. Again, there is an uneven rolling of the surface, caused by circular elevations and gutters. The bars approach a vertical position.

Even with extensive contraction the animal is not always lame; but it usually at first "points," that is, stands with the leg forewards and outwards, resting on the toe. The animal paws the litter from under the affected feet; it is lame on exit from the stable, but frequently warms out of its stilted gait and travels sound, when the enforced functional activity has brought the blood back to the compressed vessels under the heels to lubricate the dried walls. Later, the muscles of the shoulders become atrophied. (Sweeny.)

*Morbid Anatomy.*—In addition to the external alterations which are visible, and which have just been described as symptoms, there is found an atrophy of the internal structures of the foot. The frog is diminished in size, the wall is compact, thickened, and shows a discoloration of the cement; the elastic cushion is atrophied, and shows strata of fibrous tissue and yellow elastic tissue; the podophyllous laminæ are diminished in size; the third phalanx and navicular bones may be atrophied.

In "false contraction," or localized contraction of the heels, there is diminution of the transverse diameter of the heels; no increase in the height, and no increase in the thickness of their walls; this form is always acquired, is accompanied by acute lameness and fever in the heel, and is almost always due to bad shoeing.

### COMPLICATIONS.

The complications of contraction are produced by pressure, defective nutrition, unstable support, and want of functional activity, and may implicate any portion of the entire leg. *Navicular disease*, with atrophy or ulceration of the bone and interference with the synovial secretion, is the result of long-continued pressure; *corns* and ecchymoses of the podophyllous laminæ are the result of lateral pressure; *quittors* are predisposed to by the defective nutrition of the quarters; *greasy heels* find origin from the same cause; *ringbones* and *windgalls* are produced by the strains to which the bones and tendons above are subjected in the animal's attempt to alleviate the pain in the foot by false positions; contractions and degenerations of the flexor tendons occur from want of function; want of use of the leg causes atrophy of the muscles of the shoulders; uneven support of the leg causes interfering; and last but not least, if the case has been going on for some time, scars of blisters and of the hot iron and setons may be found from the fetlocks to the upper end of the scapula, which have been intended to accomplish what could have been done with a paring knife and a proper shoe.

### PROGNOSIS.

In general, the prognosis of contraction depends, to a great extent, upon the duration of the disease and the amount of atrophy of the bones and plantar cushion which has been produced. A foot which has entirely diminished in size will rarely return to its normal condition, while considerable contraction of the heels may entirely disappear, but it is wonderful what a resisting and recuperative power the foot possesses, and unexpected results are often obtained. There is no disease in which tentative treatment is so necessary before giving a definite prognosis. Trivial contraction will at times prove obstinate and become complicated with other troubles, while an excessively deformed foot and a seemingly hopeless lameness will make a rapid journey to recovery from the day of the first treatment.

DIAGNOSIS.

The diagnosis of contraction is a simple matter in itself to an anatomist and veterinarian familiar with the horse's foot, after a careful examination and comparison of first one foot and then the other, and a general estimation of the breed and character of the animal. It is again not, as a usual thing, difficult to determine just how much of the affected foot is involved; whether the whole foot is diminished in size as the result of long-continued trouble, or whether only a heel, a quarter, or some small part of the wall is contracted, the result of one or two sets of bad shoes or closely driven nails. But it is frequently a most difficult thing to determine if the contraction alone is the cause of a lameness, or if it is not complicated by other diseases; in the latter case it again becomes of the greatest importance to diagnose which is the original trouble and which the sequela. After recognizing the contraction, the shoe must be removed and examined as to its bearings and the clinching of the nails; the foot must then be pared out, and thoroughly searched for pricks, bruises, corns and any staining of the yellow line which succeeds the podophyllous tissue and limits the sole from the walls and bars. Special attention must be paid to the bars, examining if they have been pared thin or bruised. The frog will be examined by direct pressure, compression from the side, and the structure under it by counter pressure on the frog and the hollow of the pastern. The structures above will be examined for quittor, ringbone, synovitis, strains of the tendons and ligaments, and for bone troubles. Whether other troubles *exist* or do *not* exist, the shoe must be replaced in a proper manner, so as to remedy any defect of pressure or deformity as much as possible, and the animal must be re-examined cold, after warming up, and again cold. It is only in this way that the effects of the work, the presence of temporary local fever, and a proper diagnosis can be arrived at. In many cases treatment of a contraction, or of a complicating trouble, or of both, must be continued for some days before a definite diagnosis and prognosis can be given for either trouble.

TREATMENT.

The treatment of contraction is *preventive* and *curative.*

*Preventive* treatment should start with the foal by the dam's side. Winter foals and those in private hands are often forced to stand on dry floors, which bake out the moisture from the cushions of their feet before the wall and frog are fairly ready to perform their proper function, and the dried brittle mass wears off on one side and starts a contracted foot from the earliest days of the animal's life; others which have had the fortune to run in good pasture as foals, at the commencement of Winter are housed so that they have no opportunity to wear down excessive growth, and they come out in the Spring with deformed feet; others again, from some lameness, injury, or other cause, start a crooked foot, and the lateral pressure soon increases the contraction. From the time the animal is a weanling its feet should be looked after, and dressed with rasp and knife when defects in their conformation and level are found. Good, clean, dirt floors and plenty of exercise prevent dryness and brittleness of the feet; where the latter exists, either from heredity or previous carelessness, the feet should be treated so as to bring them to their normal hygroscopicity. The same rule applies to the older horse, after it has been shod. Thrushy feet are especially apt, when cured, to become excessively dry and contract. Many horses are passed as sound which would remain so if the feet were properly shod and treated from the moment of purchase, but which soon become cripples, with contraction of the feet from inattention. When an animal is to remain at rest for any time from want of use, illness, lameness of any kind, or from any reason, the feet need immediate and constant attention. At rest, especially if from a lameness, when the foot will be eased from the animal's weight, the circulation is lessened, the fluid supply to the hard coverings is diminished, the walls are exposed to the evaporating air, and the foot contracts. This must be prevented by paring down the foot, readjusting the shoes, if needed, and proper dressings to supply the deficient moisture. Carelessness is frequently shown in attempting to supply the foot with moisture. Water baths, and especially poultices, remove a certain amount of the fluid from the foot, and rot off the natural protecting varnish from the outside of the wall, and, unless they are promptly followed by other applications, are apt to render the wall dryer than it was before, and to do more harm than good. Preparations of oil are apt to become rancid and produce a rotting effect; when they are used, the foot should always be washed clean from the previous application before a fresh one is applied. Yellow wax, honey, pine tar, turpentine and heavy lubricating petroleum are among the preparations which can be used with advantage. There are also numerous inventions of sponges, fomenting pads, etc., which are of benefit, if not used constantly.

But the most important of all preventive treatment is proper shoeing. If the foot is kept on its proper level with the frog and heels bearing so as to admit of the normal elasticity of the foot, the circulation of the blood will bring the proper nutrition and the natural emollients to the surface of the kerotogenous portions of the foot, and little more will be needed except for the remedy of other pathological conditions.

*Curative Treatment.*—Most of what has been said in regard to preventive treatment is applicable to the curative treatment of contraction. When contraction has taken place the flooring and bedding of the stable must be looked to; the moisture of the media which come in contact with the feet must be regulated;

FIG. 7.

the entire hygienic surroundings of the horse much guarded, as if we had an ill animal to deal with. The foot is now to be inspected carefully and the points and amount of contraction are to be determined. It is frequently useful, especially when shoeing is to be left in the hands of a blacksmith, to take measurements of the foot in order to know just what have been gained from time to time, both in the size of the circumference of the foot at its plantar surface and coronary border, and as to the angles of the wall at toe, quarters and heels. For this purpose, and for use in instruction, I have invented an instrument, a *Podometer*, (πους, foot, and μέτρον, a measure), Fig. 7, which can be brought to bear on any part of the foot, showing the level of the sole and heels and the angles of the walls. The instrument is useful also in verifying differences of opinion which exist between the more or less experienced persons who are interested in a shoeing.

In cases of moderate contraction, with little or no twisting of the foot, it can now be leveled and the contracted portions relieved of pressure at once. In more severe cases, the alteration in shape must be nursed, a little at a time, as a too radical change may predispose to missteps, wrenches, or excessive pressure at another point, producing traumatisms at other parts. Shoes are also used which by their shape or by the addition of springs, screws, or other devices, force the heels apart; but these latter methods should be used with great caution so as not to produce rupture of the bloodvessels and complicating inflammation. The choice of shoe depends much upon the surroundings in which the horse is to be placed, if at pasture or in the stable, if at exercise only or at work, and the severity of the work; again, a case left in the veterinarian's infirmary can be treated with heroic dilatation, while that remaining with the owner must be treated with more conservatism, unless it can be seen frequently. The following are the principal methods of expansion:

*a. Bare feet;* where expansion is obtained by frog pressure; this, of course, is only applicable to animals which are to do nothing.

*b. Tips*, *slippers*, *half shoes*, *three-quarter shoes*, *semilunar shoe* or *truncated shoe*, which acts in the same way, but protects the wall from breaking (Figs. 8, 9).

*c. Bar shoes* are indicated when the frog is very much atrophied and pressure cannot be brought upon it with a plain shoe. Bar shoe as commonly made (Fig. 10); as properly made (Fig. 11).

*d. Unilateral nailing of Turner* (Fig. 12); with this system the frog and remainder of the foot has play from the toe and mamma to which the shoe is nailed.

*e. External beveling of Mayer* (Fig. 13), is supposed to throw the heels out as the weight of the animal descends on the branches.

*f. The Charlier Shoe* (Fig. 14), protects the edge of the wall below any genetic tissues and allows natural expansion.

*g. Articulated shoe* of Bracy-Clark and Vatel (Fig. 15), is not solid.

FIG. 8.[1]

FIG. 9.[1]

FIG. 10.

FIG. 11.

FIG. 12.

FIG. 13.

[1] Electros loaned by A. L. Grant, Philadelphia.

FIG. 14.

FIG. 15.

FIG. 16.

FIG. 17.

FIG. 18.

FIG. 19.[1]

[1] Electro loaned by Neverslip Horseshoe Company, Boston.

*h. Defys Expanding Shoe* (Fig. 16). This shoe can be used with most excellent results when the horse is in the hands of the veterinarian or a careful operator who will avoid too rapid expansion and be ready to combat inflammatory processes if they arise. By means of the Defys vise (Fig. 17), the amount of expansion can be regulated from day to day. The clamps turned into the lacunæ between the frog and bars should be fitted to the face of the bars exactly, and must not be allowed either to bruise the bars or press upon the frog. With this shoe I have obtained most excellent and rapid results in feet which seemed hopelessly deformed, but I always insist that I must see the case frequently.

*Expanding shoes* have also been furnished with screws set between the branches, the first by Goodwin, then one by Foures, in which the screw was set over a bar shoe, and one by Vandergrift with a fixed screw across the branches.

*Hatin* and *Steinhoff* made shoes articulated at the toe and expanded by a **V** spring (Fig. 18); later comes the *Roberge Expander*, which is held in a plain shoe and also acts directly on the bars; it only differs from Hatin's shoe in the shape of the spring.

All of these shoes have their merits, and many are adapted to special cases to which the others are not suitable, but *all* should be used with caution and replaced by a plain shoe as soon as the foot commences to assume a normal shape.

With extremely atrophied frogs, artificial pressure can be made by means of sole leather, or the "*Neverslip Horseshoe Pad*," which consists of an india rubber "frog" attached to a piece of sole leather (Fig. 19.)

FIG. 20.

It is needless to recall, except as a matter of historical interest, the *desoling* and *forcible rapid expansion* of Giordanus Ruffus, 1250 A. D.; Carlo Ruini, 1618; Soleysel and others.

Severe contraction, especially when complicated by sidebones may, however, be greatly benefited by guttering the walls of the quarters at an oblique angle to the fibres of the wall and then resorting to proper shoeing and protection of the weakened wall (Fig. 20).

### CONTAGIOUSNESS OF TUBERCULOSIS.[1]

By C. C. Lyford, M.D., D.V.S.

In accordance with a request of the State Board of Health, of Minnesota, I in May made investigations in regard to certain cases of tuberculosis. I found five sows, who had twenty-nine suckling pigs, from two to four weeks old; which, to ordinary observation, would be considered in good health. Four of these sows were Poland China—the fifth a Jersey Red. The latter showed a tendency to cough, though of so mild a nature that it might not be noticed under ordinary circumstances. All of them seemed to eat and feel well, and were in good health, excepting one of the Poland Chinas, which had had nine pigs two weeks before, and was thought to have run down on that account, showing no other signs of disease.

At first it seemed a question as to how it had originated, but upon investigation, I found that there had been from twenty-two to twenty-five last Spring pigs, all apparently in good health up to January 1st, prior to which time they had run together with fat cattle around farm buildings. During the latter part of December six pigs were sent to their farm, some four miles away. The others still ran with cattle, as before. About the middle of January a steer, about five years old, became emaciated, the bowels being very loose for some weeks, and a cough having appeared. The animal was almost unable to get up, consequently was killed and drawn into a back yard for the pigs to eat. Upon examination of the steer, the foreman of the farm reported that his lungs, liver, spleen and kidneys were studded with indications of tuberculi.

During the month of February from three to five farrows became weak, especially across the back, and either died or were killed. The foreman reported to have opened each of them, and in every case found lungs, liver and kidneys with the same markings as those in the steer. Nothing more was thought of these

[1] Read before the twenty-seventh annual meeting of the United States Veterinary Medical Association, at Chicago, September 17th, 1890.

cases until recently, when eight hogs, apparently in good condition, were received by De Witt & Sons, Minneapolis, which were killed and dressed, and in each and every case the lungs, spleen and kidneys were more or less affected with tuberculi. These were reported, having been examined by Inspectors Davies, De Witt and Schwartzkopff. Specimens from the above cases I have for investigation. Some weeks prior to receiving the above shipment, six hogs were brought from the other farm—being from the same litter, as before said—which were found in good health, and passed inspection.

I might here state that I found a seven-year-old cow on the first farm, which had been farrow the past year, being greatly emaciated and having a severe cough. This the owner consented to have killed for investigation. This was done, and I found her a mass of tuberculi. Her last calf was killed, when a few weeks old, for veal, and the cow—said to have taken cold at the time of calving, which took place in a severe storm—was dried up, and has since been in poor health. No other case of the kind has been known to have been on the farm, excepting one, some three or four years ago. It might be well to state that the pigs received little or no milk from the cows on the farm—there being several calves, which are said to have taken it all. Some twenty this Spring calves were in the yard, being from Hereford bulls owned on the premises—the bulls being apparently healthy and from two to three years old.

## Figures and Tables

**Fig. 1. f1:**
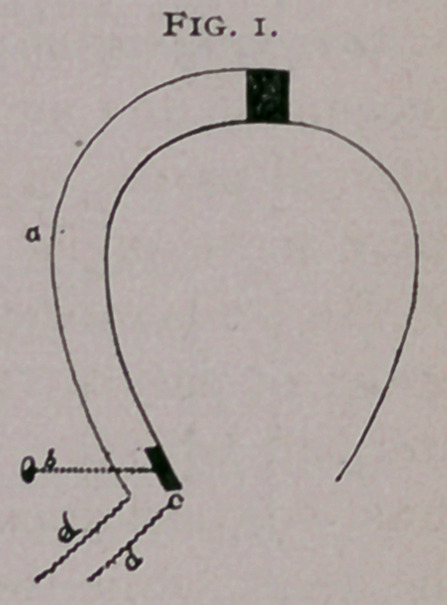


**Fig. 2. f2:**
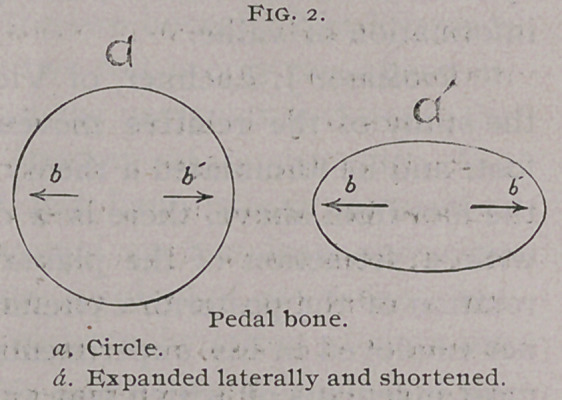


**Fig. 3. f3:**
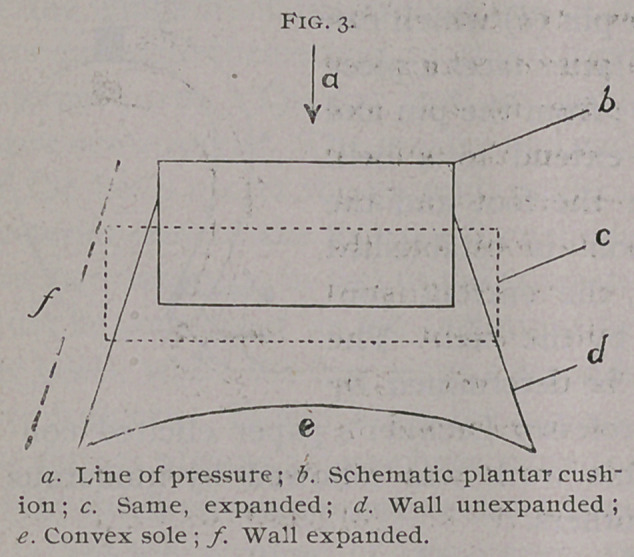


**Fig. 4. f4:**
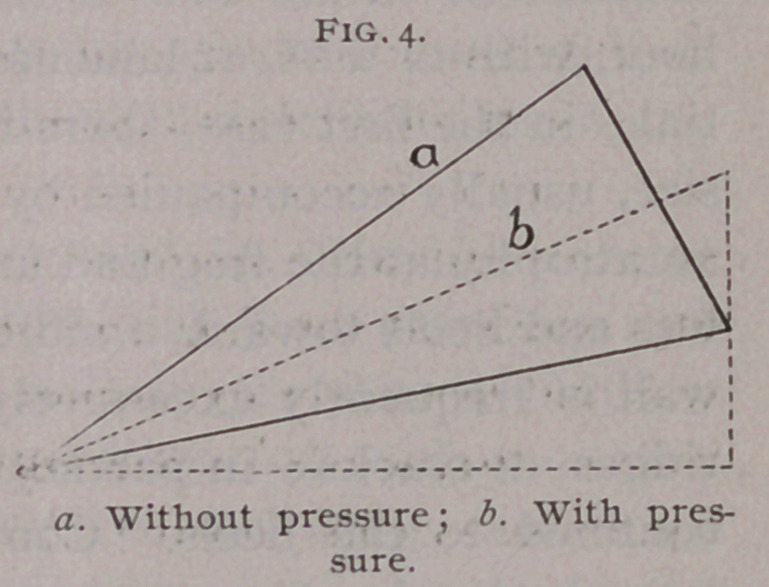


**Fig. 5. f5:**
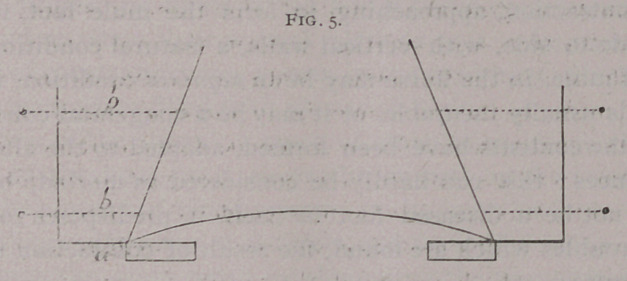


**Fig. 6. f6:**
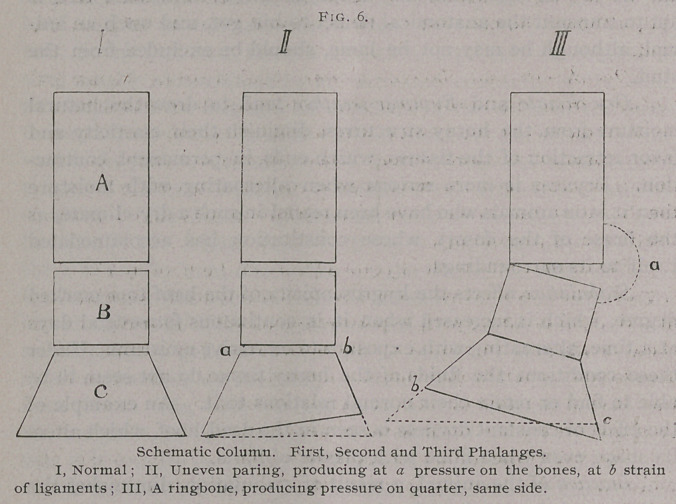


**Fig. 7. f7:**
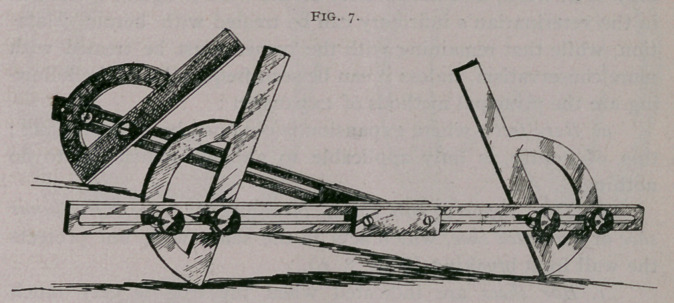


**Fig. 8. f8:**
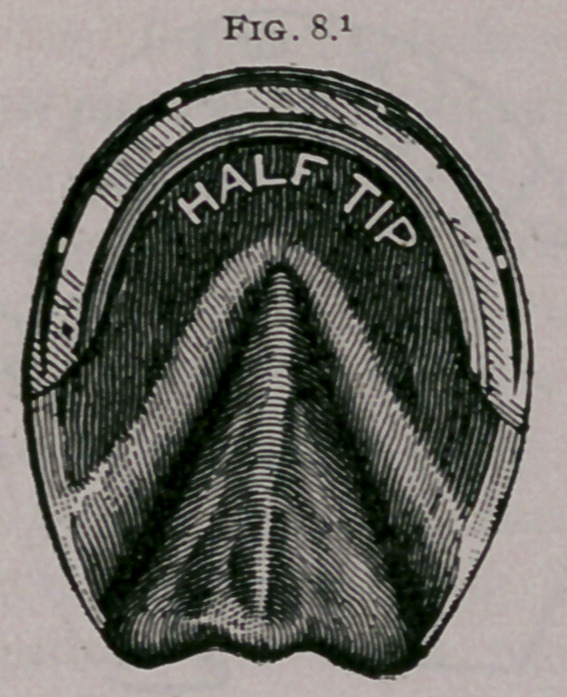


**Fig. 9. f9:**
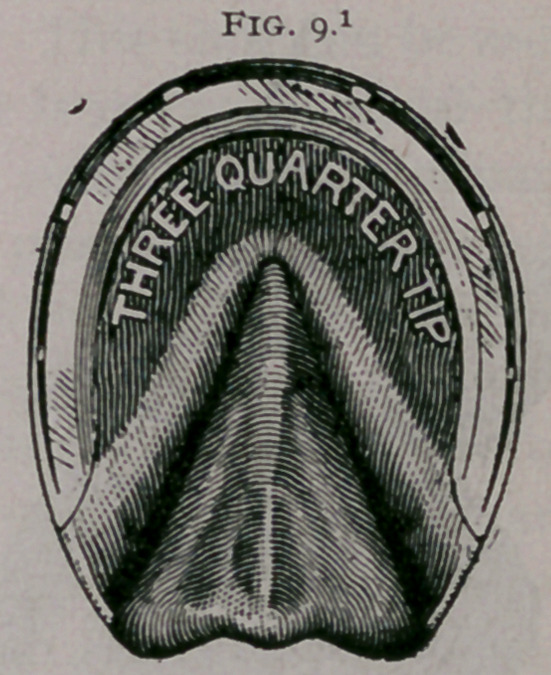


**Fig. 10. f10:**
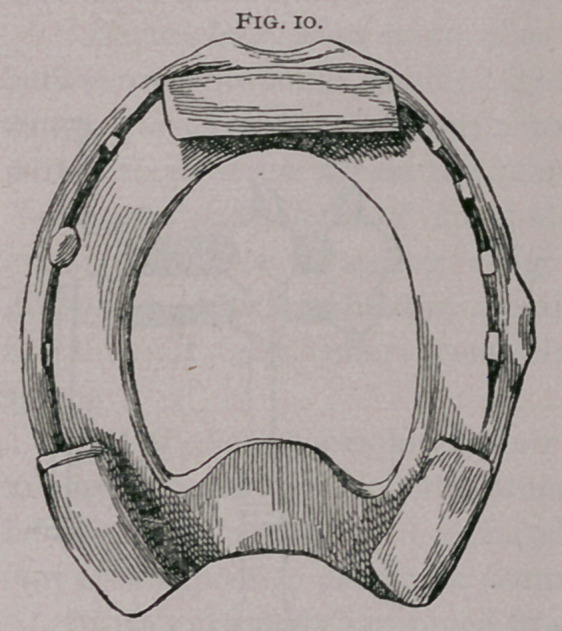


**Fig. 11. f11:**
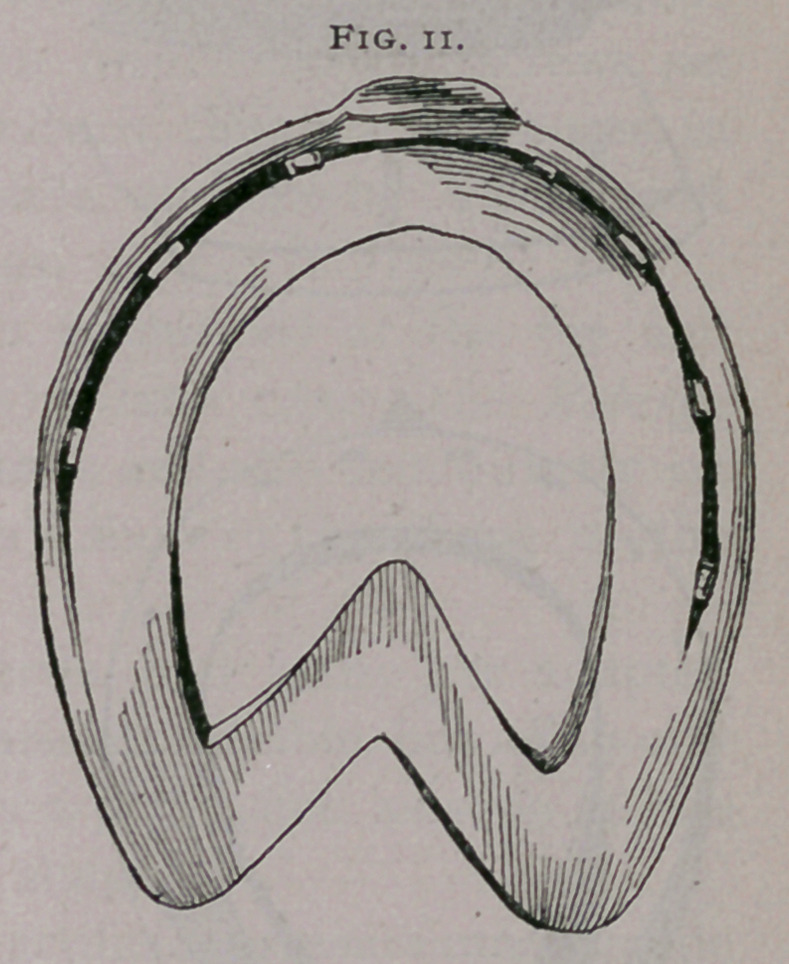


**Fig. 12. f12:**
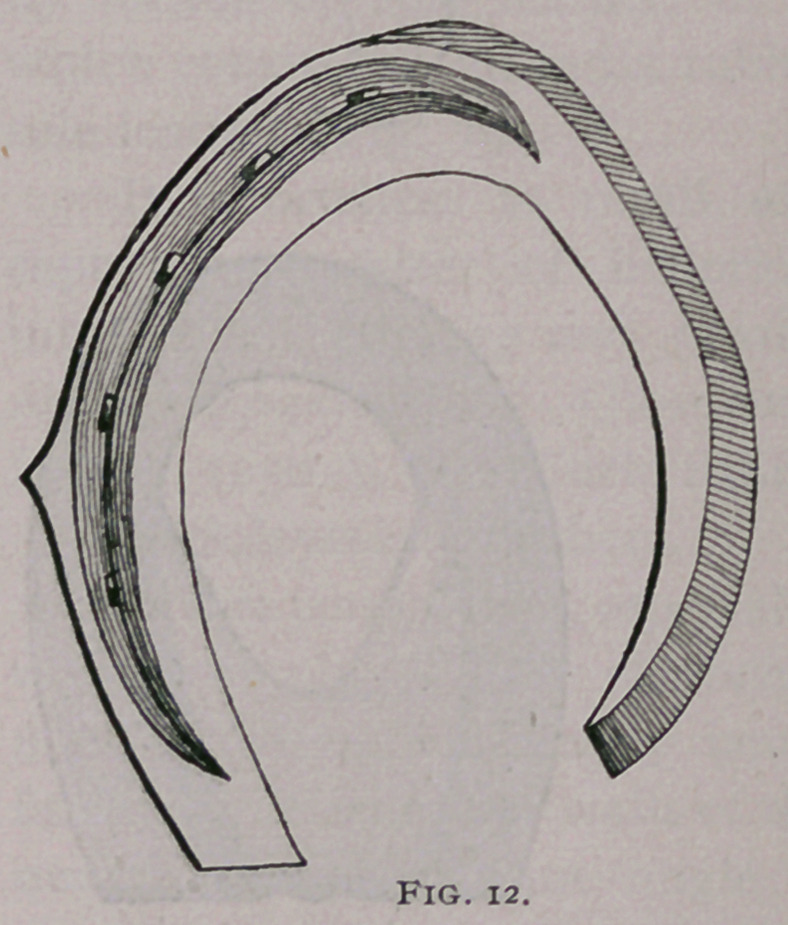


**Fig. 13. f13:**
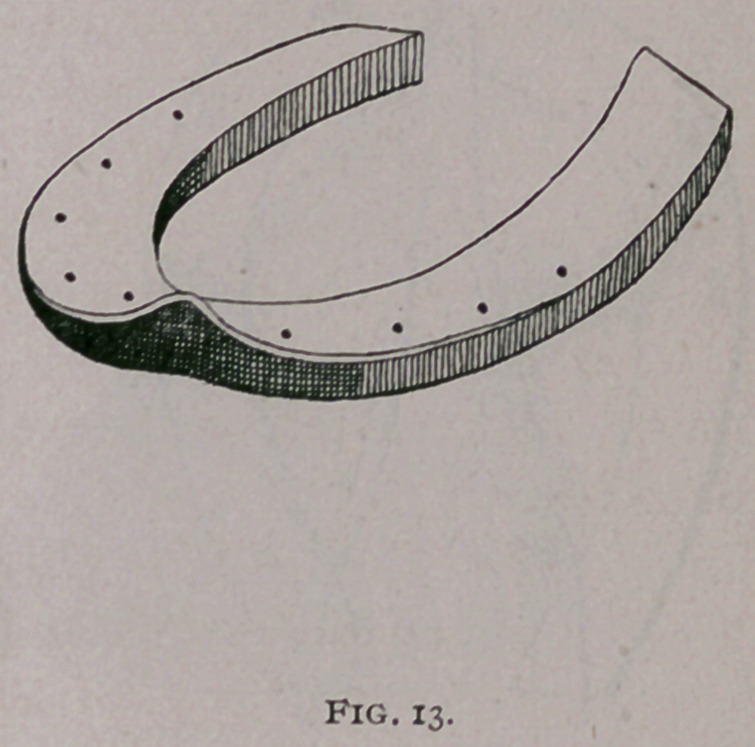


**Fig. 14. f14:**
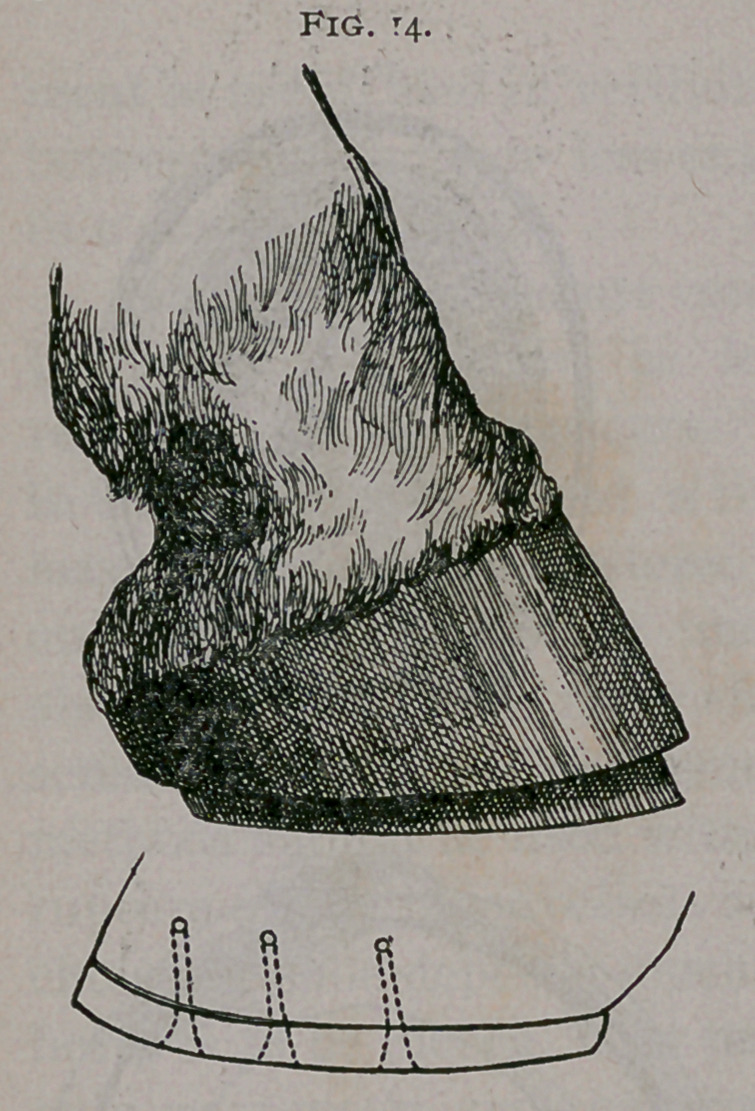


**Fig. 15. f15:**
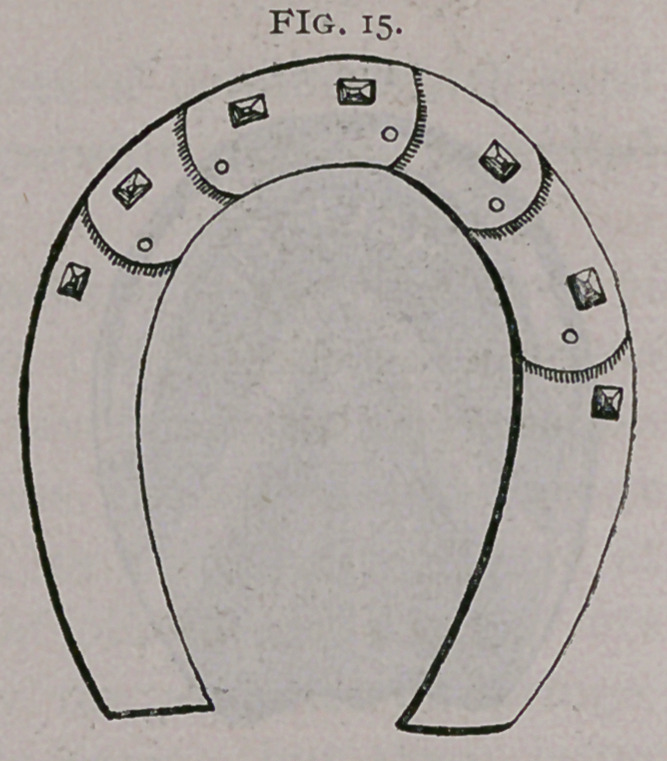


**Fig. 16. f16:**
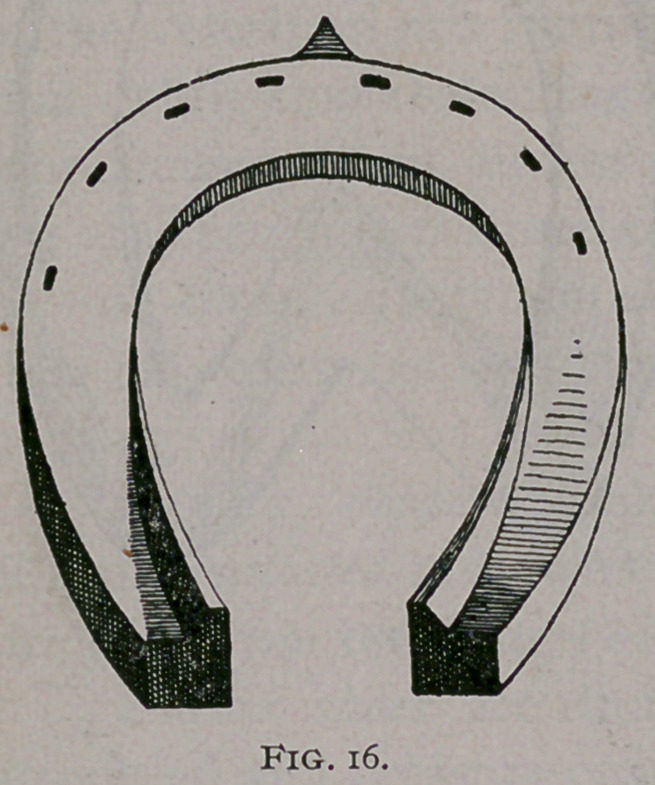


**Fig. 17. f17:**
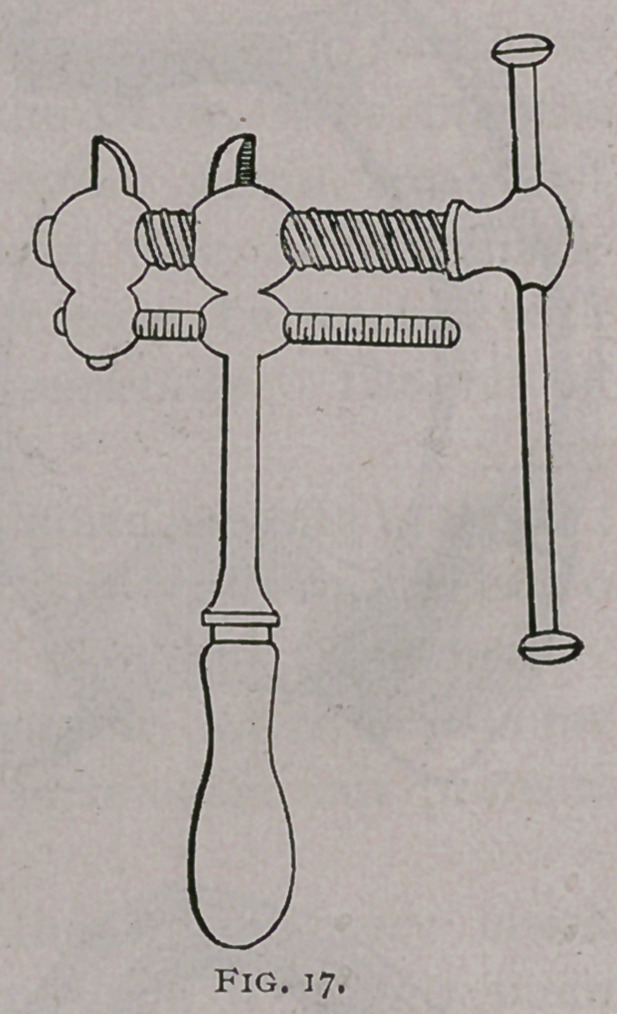


**Fig. 18. f18:**
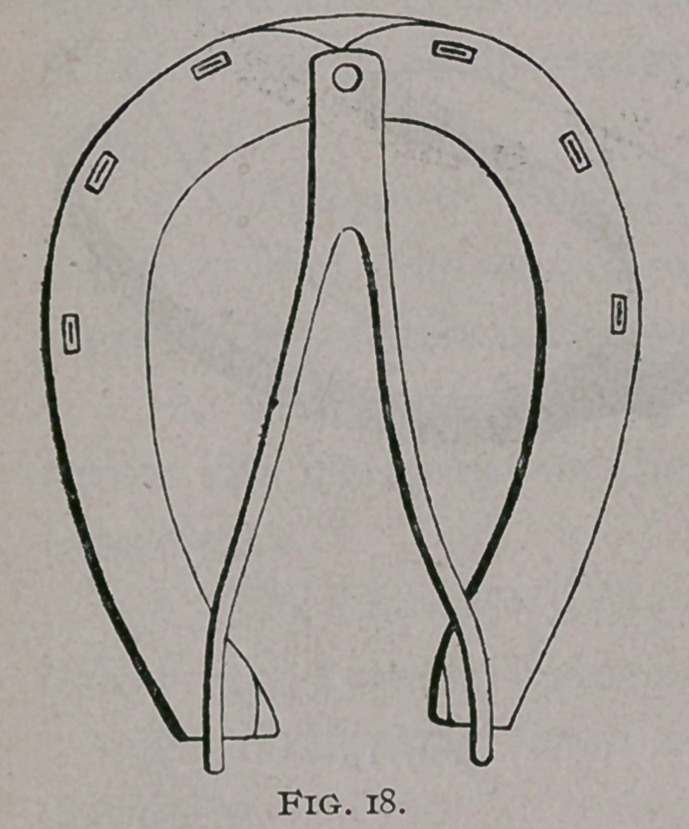


**Fig. 19. f19:**
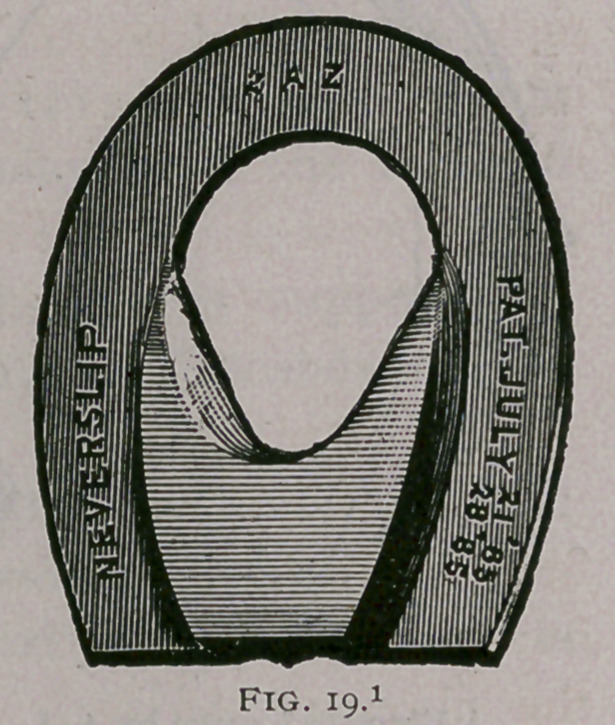


**Fig. 20. f20:**